# T Cell and Dendritic Cell Abnormalities Synergize to Expand Pro-Inflammatory T Cell Subsets Leading to Fatal Autoimmunity in B6.NZBc1 Lupus-Prone Mice

**DOI:** 10.1371/journal.pone.0075166

**Published:** 2013-09-20

**Authors:** Nafiseh Talaei, Yui-Ho Cheung, Carolina Landolt-Marticorena, Babak Noamani, Timothy Li, Joan E. Wither

**Affiliations:** 1 Arthritis Centre of Excellence, Division of Genetics and Development, Toronto Western Research Institute, University Health Network, Toronto, Ontario, Canada; 2 Department of Immunology, University of Toronto, Toronto, Ontario, Canada; 3 Department of Medicine, University of Toronto, Toronto, Ontario, Canada; 4 Division of Rheumatology, University Health Network, Toronto, Ontario, Canada; McGill University Health Center, Canada

## Abstract

We have previously shown that B6 congenic mice with a New Zealand Black chromosome 1 (c1) 96-100 cM interval produce anti-nuclear Abs and that at least two additional genetic loci are required to convert this subclinical disease to fatal glomerulonephritis in mice with a c1 70-100 cM interval (c1(70-100)). Here we show that the number of T follicular helper and IL-21-, IFN-γ-, and IL-17-secreting CD4^+^ T cells parallels disease severity and the number of susceptibility loci in these mice. Immunization of pre-autoimmune mice with OVA recapitulated these differences. Differentiation of naïve T cells *in-vitro* under polarizing conditions and *in-vivo* following adoptive transfer of OVA-specific TCR transgenic cells into c1(70-100) or B6 recipient mice, revealed T cell functional defects leading to increased differentiation of IFN-γ- and IL-17-producing cells in the 96-100 cM and 88-96 cM intervals, respectively. However, in*-vivo* enhanced differentiation of pro-inflammatory T cell subsets was predominantly restricted to c1(70-100) recipient mice, which demonstrated altered dendritic cell function, with increased production of IL-6 and IL-12. The data provide support for the role of pro-inflammatory T cells in the conversion of subclinical disease to fatal autoimmunity and highlight the importance of synergistic interactions between individual susceptibility loci in this process.

## Introduction

Systemic Lupus Erythematosus (SLE) is a generalized autoimmune disease characterized by the production of autoantibodies, particularly those directed against nuclear antigens, which form immune complexes that deposit in tissues. Studies of SLE in humans and lupus-prone mice indicate that multiple genetic polymorphisms affecting diverse immune populations interact with each other to produce the lupus phenotype. Among these populations are T helper (Th) cells. Although early studies demonstrated a predominant role for Th1 cells in lupus, several recent studies suggest that two other pro-inflammatory Th cell subsets, T follicular helper (Tfh) and Th17 cells, are also pathogenic [1].

Tfh cells are a distinct subset of Th cells that provide help for antigen specific B cell responses in the context of germinal centers (GC) and produce high levels of IL-21 [2,3]. A potential role for this population in the pathogenesis of lupus was first suggested by the observation that lupus-prone mice with a homozygous point mutation in the *Roquin* gene, demonstrated expansion of their Tfh population, and subsequently supported by demonstration of similar expansions in MRL^lpr^ and BXSB/Yaa lupus-prone mice [4].

Although Th17 cells are defined by their IL-17 production, they produce a variety of other cytokines including IL-21, IL-22, TNF-α, IL-6 and IL-9 [5]. Expansion of this population has been demonstrated in several lupus-prone mouse strains, including (New Zealand Black (NZB) x SWR) F_1_, TNF receptor 1 and 2 gene-deleted New Zealand Mixed 2328, and BXD2 mice [6,7,8]. Notably, introduction of a null gene for the IL-17A receptor onto the BXD2 background significantly attenuated production of IgG autoantibodies and nephritis [8]. Despite compelling evidence that Tfh and Th17 cells play a central role in lupus pathogenesis, the genetic basis leading to the aberrant activation of these cell populations remains unknown.

To characterize the immunologic abnormalities that promote lupus, our laboratory has produced a series of congenic mouse strains with homozygous NZB chromosomal intervals crossed onto the non-autoimmune C57BL/6 (B6) background. In previous experiments we showed that mice with a NZB c1 interval extending from 70-100 cM (c1(70-100)) develop a severe lupus phenotype, with high titers of anti-dsDNA Abs and glomerulonephritis (GN), leading to death of ~40% of the mice by 8 months of age. This phenotype appeared to result from at least 3 genetic loci, as indicated by progressively attenuated disease in mice with NZB c1 intervals extending from 88- or 96-100 cM [9]. Here we show that the disease severity in these mice parallels the expansion of pro-inflammatory T cell subsets, specifically Th1, Th17, and Tfh cells. We further demonstrate that this expansion can be recapitulated following immunization of pre-autoimmune mice with an exogenous antigen. This T cell skewing results from a combination of immune cell functional abnormalities in congenic mice that localize to different regions within the c1 70-100 interval. Naïve T cell functional abnormalities that lead to expansion of IFN-γ- and IL-17- producing cells localized to the 96-100 and 88-96 intervals, respectively, whereas dendritic cell (DC) functional abnormalities that promote expansion of all the pro-inflammatory T cell subsets localized to the 88-96 and 70-88 intervals. Notably, altered DC function appeared to play a critical role in this expansion, because in the absence of DC abnormalities, minimal expansion of pro-inflammatory T cell subsets was seen. Our findings provide insight into how individual susceptibility loci, which alone produce modest changes in immune function, interact synergistically to profoundly alter immune function leading to severe clinically relevant autoimmune disease.

## Results

### Expansion of pro-inflammatory CD4^+^ T cell subsets in NZB c1 congenic mice

**Figure 1:**
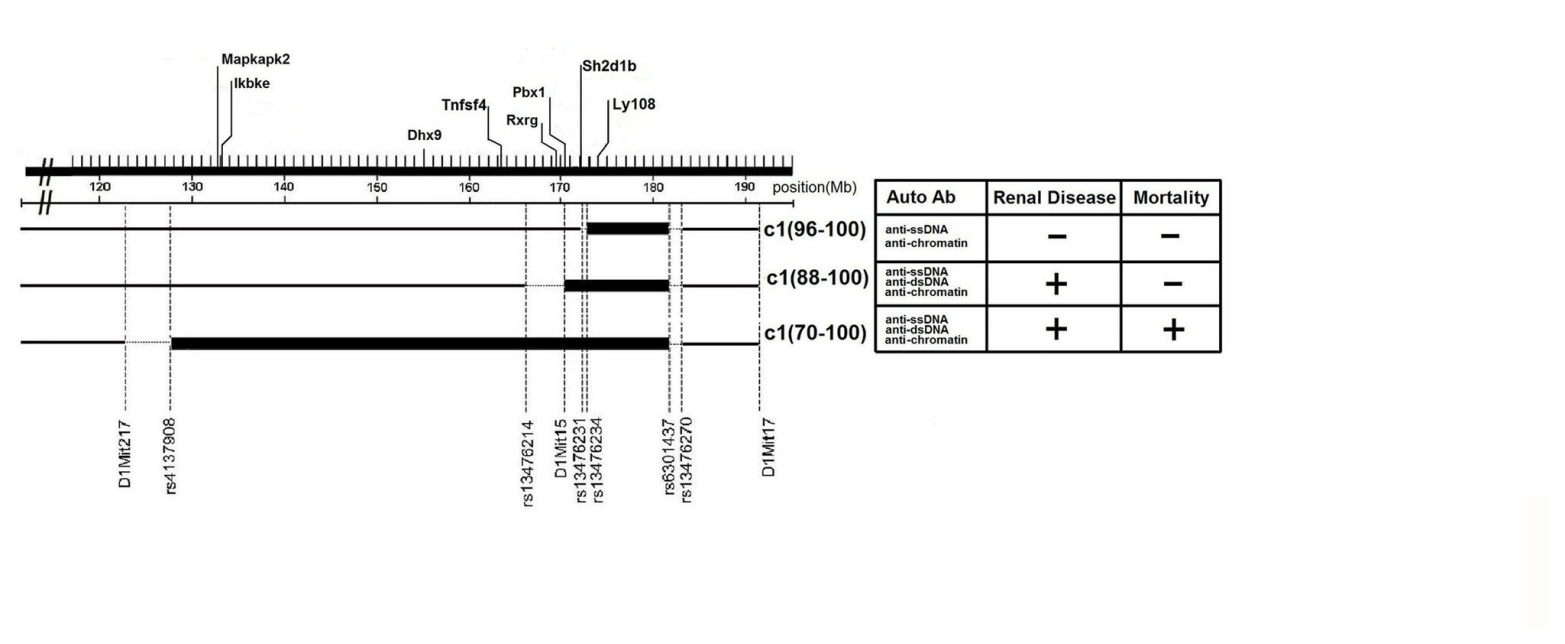
Genetic map of the c1 congenic mouse strains studied. Thick and thin lines denote NZB and B6 regions, respectively. Dashed lines indicate regions of undefined origin. Polymorphic microsatellite markers and single nucleotide polymorphism (SNP) markers were used to discriminate between NZB and B6 DNA at the termini of the regions according to the NCBI 2007 (m37 release) mouse genome assembly (www.ensembl.org). Potential candidate genes within the interval are indicated above the chromosomal map. Phenotypic features of NZB c1 congenic mouse strains are shown to the right of the c1 congenic mice genetic map [9].

**Figure 2:**
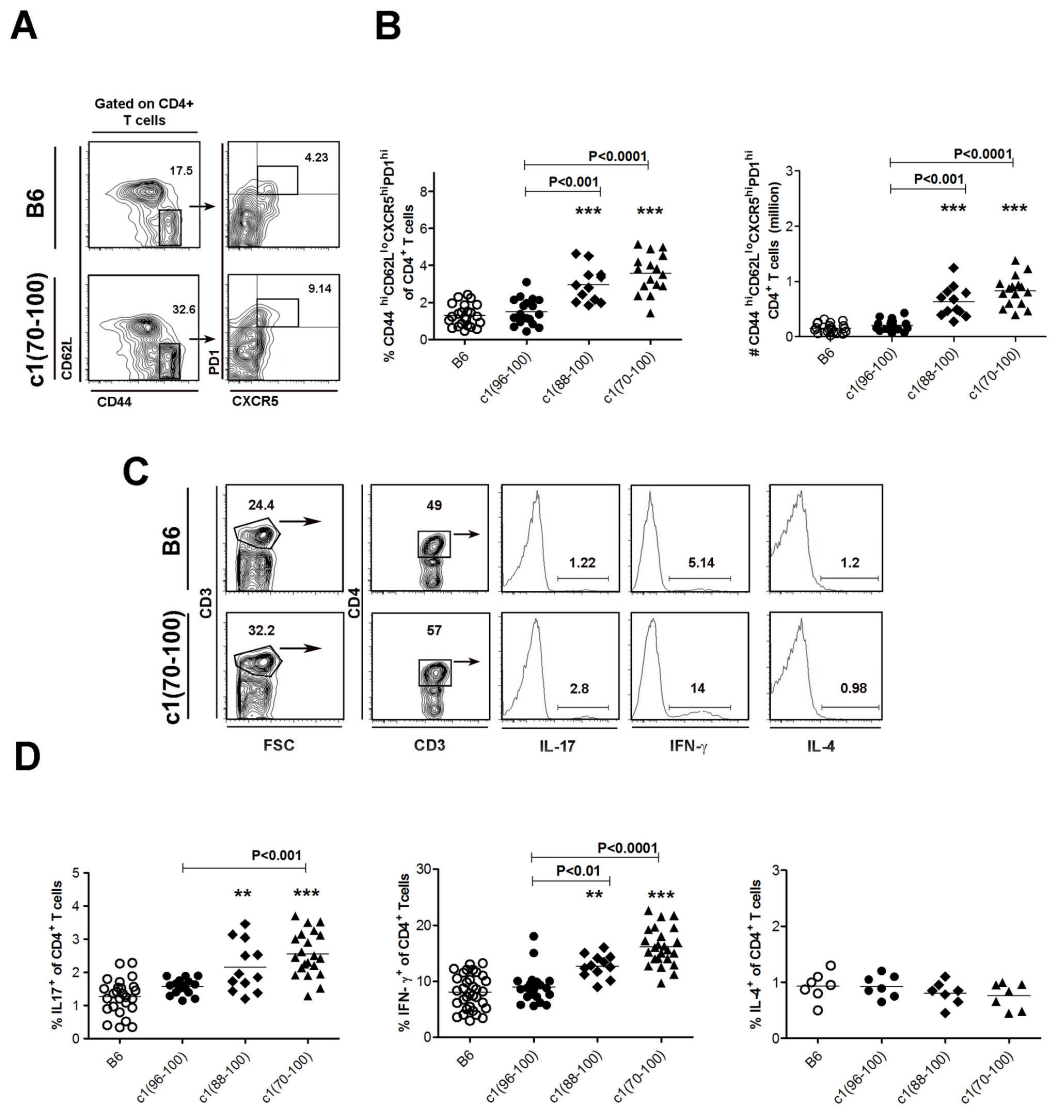
Expansion of Tfh, Th17 and Th1 cell subsets in c1 congenic mice. (A) Splenocytes from 4-mo-old mice were stained to assess the proportion of Tfh (CD4^+^CD44^hi^CD62L^lo^CXCR5 ^hi^PD1^hi^) cells. Representative contour plots from B6 and c1(70-100) mice. Thick boxes denote the regions that were used to identify Tfh cells. Cells shown in the right panels were gated on the regions shown in the left panels. (B) Scatter plots showing the proportion of Tfh cells within the CD4^+^ T cell subset and absolute number of splenic Tfh cells. (C) Representative contour plots and histograms from flow cytometry analysis of IL-17-, IFN-γ-, and IL-4-expressing CD4^+^ T cells in B6 and c1(70-100) mice. Splenocytes were stimulated with PMA and ionomycin in the presence of GolgiStop for 4 h, and then fixed, stained with anti-CD3 and -CD4, permeabilized, and stained with anti-cytokine Ab. Thick lines outline the regions used to gate CD4^+^CD3^+^ T cells. For histograms, the percentage of cells staining positively for each cytokine is indicated. (D) Scatterplots showing the percentages of cytokine-producing cells as a proportion of the CD4^+^ T cell population. Horizontal lines indicate the mean of each group examined. Significance levels were determined by one-way ANOVA with Dunns’ post-test. The *p* values for significant differences between B6 and congenic mouse strains are shown with **p<0.01, ***p<0.001. Bars with *p* values above denote significant differences between congenic strains.

B6 congenic mice with NZB c1 intervals extending from 96-100 cM (172.8-183.0 Mb; c1(96-100)), 88-100 cM (170.3-183.0 Mb; c1(88-100)) or 70-100 cM (126.6-183.0 Mb; c1(70-100)) demonstrate progressively more severe disease with increasing length of the c1 interval (Figure 1). Since increases in the number and size of GC paralleled disease severity in these mice, we postulated that changes in Th cell number/function were producing these differences. To address this possibility, Th cell subsets were examined in 4-mo-old B6 and congenic mice, using flow cytometry. As shown in Figure 2A& B, the proportion and number of Tfh cells (gated as CD4^+^CD44^hi^CD62L^lo^CXCR5 ^hi^PD1^hi^) was significantly increased in c1(88-100) and c1(70-100) mice, whereas the level of these cells in c1(96-100) mice was similar to B6 mice. Consistent with the increases in Tfh, and our previous findings, there was a trend to increased proportions of GC B cells in all three congenic mouse strains with the greatest increase seen in c1(70-100) mice (Figure S1A& B). The expansion of Tfh was further confirmed by immunofluorescence microscopy where increased numbers of Tfh cells were seen in the GC of c1(88-100) and c1(70-100) mice (Figure S1C& D).

To examine the other Th subsets, cytokine-producing CD4^+^ T cells were quantified by flow cytometry following stimulation of freshly isolated splenocytes for 4 hrs with PMA and ionomycin, and intracellular staining for IL-4, IFN-γ, and IL-17 (Figure 2C). There were no differences between strains in the proportion of IL-4 producing cells, but a trend to a progressive increase in IFN-γ and IL-17 producing T cells with increasing size of the NZB c1 interval was seen (Figure 2D). Similar findings were obtained when CD4^+^ T cells were stimulated *in-vitro* with anti-CD3 and -CD28 Abs, and secretion of various cytokines was quantified in the supernatants (Figure S2). While there was no significant difference between the mouse strains in the production of IL-2 and IL-4, there was a progressive increase in the secretion of IFN-γ, IL-17, and IL-21 that correlated with increasing length of the c1 interval.

To further define the CD4^+^ T cell populations secreting these cytokines, intra-cellular cytokine levels were examined in cells stained with anti-CD3, -CD4, -CXCR5, and -PD1 to permit discrimination between Tfh (CD4^+^CXCR5 ^hi^PD1^hi^) and conventional CD4^+^ T cells (including Th17 and extrafollicular T cells). This revealed that the increase in IL-21 and IFN-γ secreting cells observed in c1 mice results from increases in the numbers of both Tfh and conventional CD4^+^ T cells that secrete these cytokines (Figure S3A& B), which positively correlated with the number of NZB genetic loci. A significant proportion of the IL-21 secreting cells also secreted IFN-γ (20-40% Tfh, 40-60% conventional), and conversely IFN-γ secreting cells also secreted IL-21 (40-60% Tfh, 30-50% conventional), with the proportion of co-secretors paralleling the length of NZB interval in congenic mice (Figure S3A and data not shown).

The majority of IL-17 secreting cells were seen in the conventional CD4^+^ T cell population (Figure S3B), with ~5% of the cells also secreting IFN-γ and 80% of cells secreting IL-21 in all mouse strains examined (Figure S3A and data not shown). Consistent with the flow cytometry findings, the majority of IL-17 secreting cells were seen within the T cell zone and the number of these cells was increased in c1(88-100) and c1(70-100) mice (Figure S3C& D).

### Intrinsic skewing of the immune system towards increased generation of Tfh, Th17 and Th1 cell subsets in c1 congenic mice

**Figure 3:**
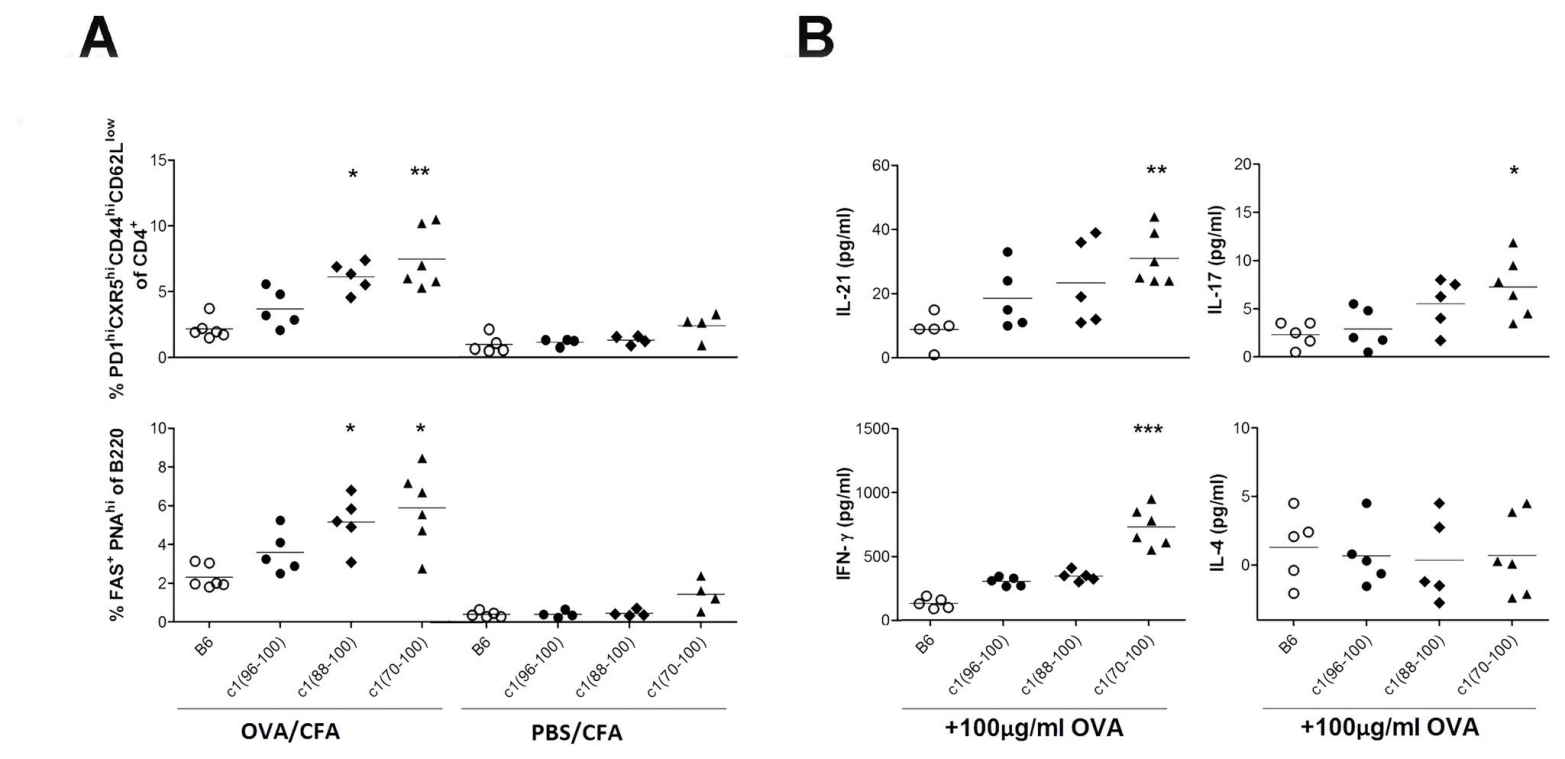
Enhanced differentiation of pro-inflammatory T cell subsets in c1 congenic mice following OVA immunization. 8-wk-old mice were injected i.p. with OVA or PBS in CFA. The proportions of splenic Tfh cells (CD4^+^CD44^hi^CD62L^lo^CXCR5 ^hi^PD1^hi^) and splenic GC B cells (B220 ^+^ Fas ^+^ PNA^hi^) were determined by flow cytometry 2 wks later. (A) Scatterplots showing the proportion of Tfh and GC B cells as a proportion of the CD4^+^ T cell and B220^+^ B cell populations, respectively. (B) Scatterplots showing the amount of cytokine produced by OVA-primed splenocytes re-stimulated *in-vitro* with OVA for 72h. Assays were performed in triplicate and the levels of secreted cytokines measured by ELISA or cytokine bead array (see Methods). Each data point represents the mean of the triplicate with background cytokine production in the absence of antigen subtracted. Horizontal lines indicate the mean of each group examined. Significance levels were determined by one-way ANOVA with Dunns’ post-test. The *p* values for significant differences between B6 and congenic mouse strains are shown with *p<0.05, **p<0.01, ***p<0.001.

To determine whether the increased production of IL-21, IL-17, and IFN-γ in c1 congenic mice was a consequence of the breakdown in tolerance to nuclear antigens, or resulted from intrinsically altered immune function leading to skewed Tfh, Th17 and Th1 development, we investigated the immune response to OVA as a representative exogenous antigen. Young pre-autoimmune 8-wk-old B6 and c1 congenic mice were immunized i.p. with OVA emulsified in CFA, using PBS emulsified in CFA as a control. The mice were sacrificed 14 days later and the proportions of various T cell subsets and GC B cells were examined. Consistent with our previous results (Figure 2 and Figure S1), there was a progressive increase in the proportion of Tfh and GC B cells corresponding to increasing size of the NZB c1 interval, following OVA-CFA immunization (Figure 3A). No significant differences were observed with PBS-CFA immunization. To assess the cytokine profile of the OVA-specific T cells, splenocytes isolated from OVA-primed mice were re-stimulated *in-vitro* with OVA for 72 h. Cytokine levels were measured in tissue culture supernatants and the amount of OVA-specific cytokine production was determined by subtracting cytokine production in the absence of OVA. As seen in 4-mo-old unimmunized mice, there were progressive increases in IFN-γ, IL-17, and IL-21 production with increasing length of the NZB c1 interval (Figure 3B). Thus, the immune system in c1 congenic mice appears to be intrinsically skewed toward increased production of Th1, Th17, and Tfh cytokines, regardless of the specificity of the antigen.

### Altered T cell differentiation in c1 congenic mice results from defects affecting T and non-T cell function

**Figure 4:**
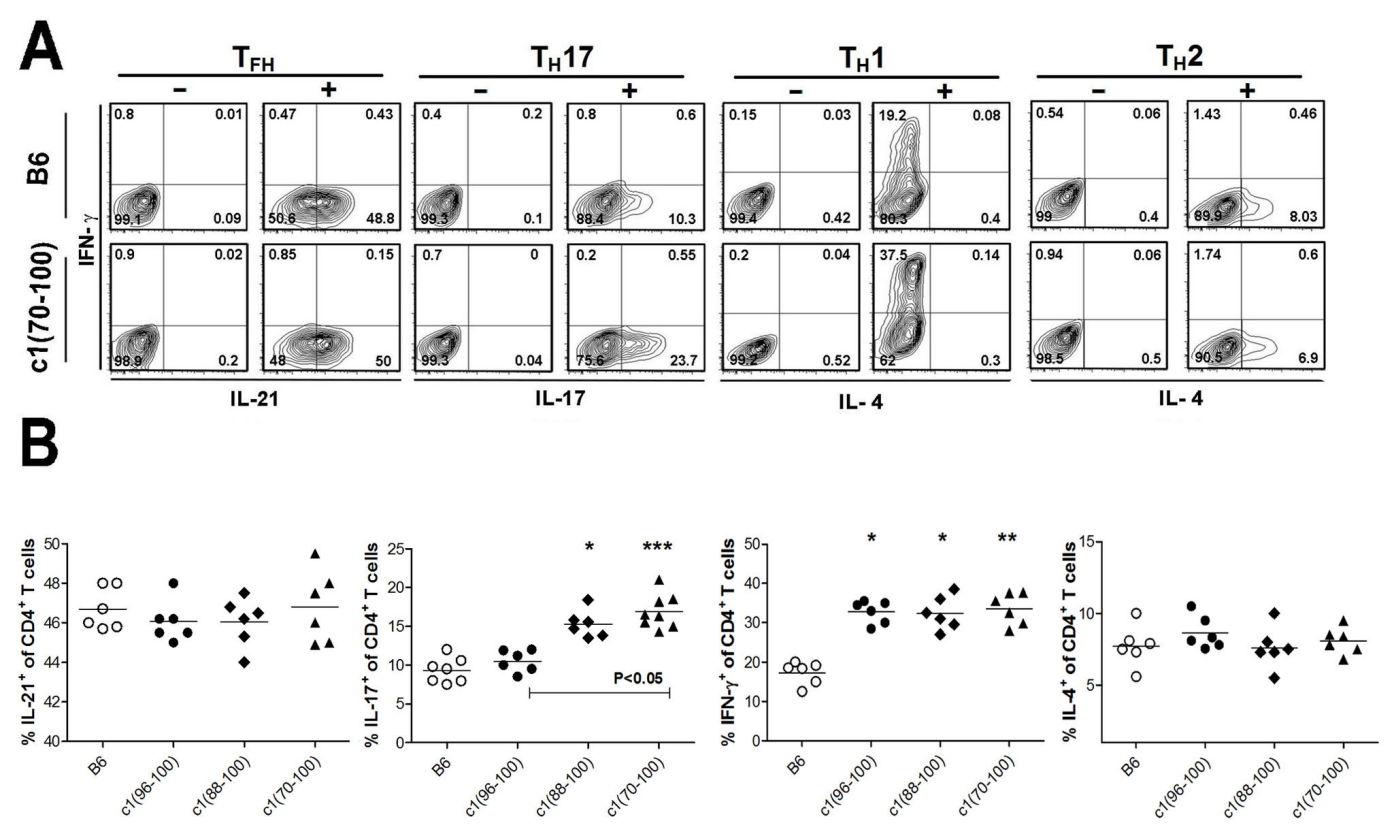
Increased differentiation of naïve CD4^+^ T cells from c1 congenic mice to Th17 and Th1 cells *in-vitro*. Naïve T cells from 8-wk-old mice were stimulated under Th0, Th1, Th2, Th17, and IL-21-producing polarizing conditions and cytokine production quantified 5 days later by flow cytometry (see Methods). (A) Representative contour plots gated on CD3^+^CD4^+^ T cells from B6 and c1(70-100) mice. For each polarizing condition, plots for relevant cytokine production under Th0 conditions (-) and polarizing conditions (+) are shown. The quadrants used to define positively and negatively staining cells are indicated. (B) Scatterplots showing the percentage of T cells that are IL-21-producing (Tfh), Th17, Th1 and Th2 cells, under relevant polarizing conditions. Horizontal lines indicate the mean for each population examined. Significance levels were determined by one-way ANOVA with Dunns’ post-test. The *p* values for significant differences between B6 and congenic mouse strains are shown with *p<0.05, **p<0.01, ***p<0.001. Bars with *p* values above denote significant differences between congenic strains.

To determine the immune defects that lead to the increased differentiation of CD4^+^ T cells into Th1, Th17 and Tfh cells in c1 congenic mice, several approaches were used. In the first approach, naïve T cells from the spleens of 8-wk-old pre-autoimmune mice were isolated and induced to differentiate into various T cell subsets using cocktails of cytokines and mAbs (see Materials and Methods). Under Th0 conditions there was minimal differentiation of either B6 or c1 congenic T cells into IL-21- (< 0.21%), IL-17- (<0.12%), IFN-γ- (<0.82%), or IL-4- (<0.41%) secreting cells with similar levels seen for all mouse strains (Figure 4A and data not shown). In contrast, under Th1-inducing conditions, all c1 congenic mice demonstrated increased differentiation to IFN-γ-secreting cells compared to B6 mice (Figure 4B), suggesting that a genetic locus in the NZB c1 96-100 cM interval promotes differentiation of this cell subset. Using Th17-inducing conditions, both c1(88-100) and c1(70-100) naïve T cells demonstrated equivalently increased differentiation to IL-17-producing cells compared to B6 or c1(96-100) T cells. Thus, a genetic locus located within the NZB c1 88-96 interval alters T cell function to promote IL-17 secretion. In contrast, similar proportions of Th2 and IL-21-producing cells were seen for all mouse strains tested under their respective cytokine inducing conditions, suggesting that the increased proportions of IL-21 producing cells seen *in-vivo* in c1 congenic mice do not arise from a T cell functional defect.

**Figure 5:**
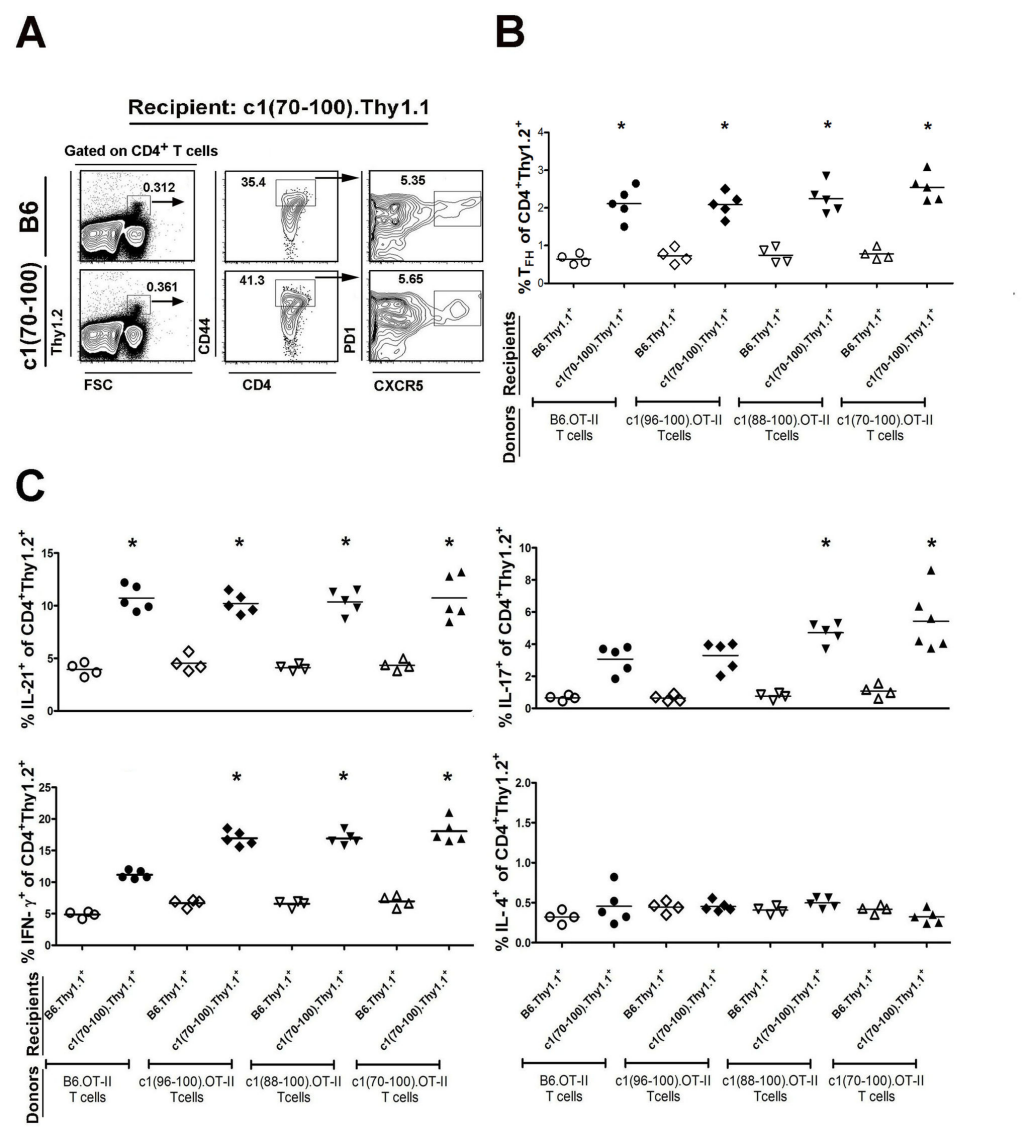
Intrinsic T cell functional defects together with altered environmental cues promote the enhanced differentiation of OVA-specific T cell subsets in congenic mice. Naïve T cells from OT-II TCR Tg mice were transferred into pre-autoimmune B6.Thy1.1 or c1(70-100). Thy1.1 mice, that were subsequently immunized with OVA in CFA. Mice were sacrificed 2 wks later and the proportion of transferred T cells differentiating to various T cell subsets was examined by flow cytometry. (A) Representative contour plots following transfer of B6 or c1(70-100) OT-II cells into c1(70-100). Thy1.1 mice. Transferred cells were identified by staining the splenocytes from recipient mice with anti-Thy1.2 mAb. Tfh cells were identified by gating on the CD4^+^CD44^hi^PD1 ^hi^CXCR5^hi^ cells (indicated by boxed regions) within this subset. Cytokine-producing cells were identified as outlined in Figures 1 and 2, and the Methods. Scatter plots of the proportion of (B) Tfh and (C) cytokine-producing cells within the transferred T cell population. The open and closed symbols represent cells transferred into B6 or c1(70-100) recipient mice. Horizontal lines indicate the mean of each group examined. Significance levels were determined by one-way ANOVA with Dunns’ post-test. The *p* values for significant differences between B6 and congenic mouse strains are shown with *p<0.05.

The second approach used to examine the altered T cell differentiation in c1 congenic mice was adoptive transfer of B6 or congenic T cells into B6 or congenic recipients in a reciprocal fashion. To facilitate these investigations, an OT-II TCR transgene (Tg) with specificity for OVA/A^b^, was crossed onto the various mouse backgrounds. Naïve CD4^+^ T cells were then purified from the spleens of young 8-wk-old OT-II TCR Tg B6 and c1 congenic mice, and injected into the tail vein of 8-wk-old B6.Thy1.1 or c1(70-100). Thy1.1 mice. Mice were then immunized with OVA and the differentiation of naïve OT-II T cells into various Th cell subsets determined by flow cytometry, gating on the transferred Thy1.2^+^ population (Figure 5A). These results confirmed the *in-vitro* Th differentiation results, showing that the enhanced IFN-γ- and IL-17-, but not IL-21-, secreting cell differentiation arises in part from intrinsic T cell defects localizing to the NZB c1 96-100 and 88-96 intervals, respectively (Figure 5B& C). However, there was also an important role for the environment in the increased differentiation that was observed, because OT-II T cells from all of the mouse strains demonstrated enhanced differentiation to Tfh, Th1, Th17, and IL-21-secreting populations when transferred into c1(70-100). Thy1.1 mice. Indeed, only minimal non-significant increases in the proportion of IFN-γ- and IL-17-secreting cells for the relevant c1 congenic T cells were seen upon adoptive transfer into B6 mice. This finding suggests that the increased differentiation of these T cell subsets in c1 congenic mice is critically dependent upon cellular and/or cytokine cues that are not provided by the B6 environment.

### DC from c1(88-100) and c1(70-100) mice demonstrate altered function that promotes differentiation of pro-inflammatory T cell subsets

**Figure 6:**
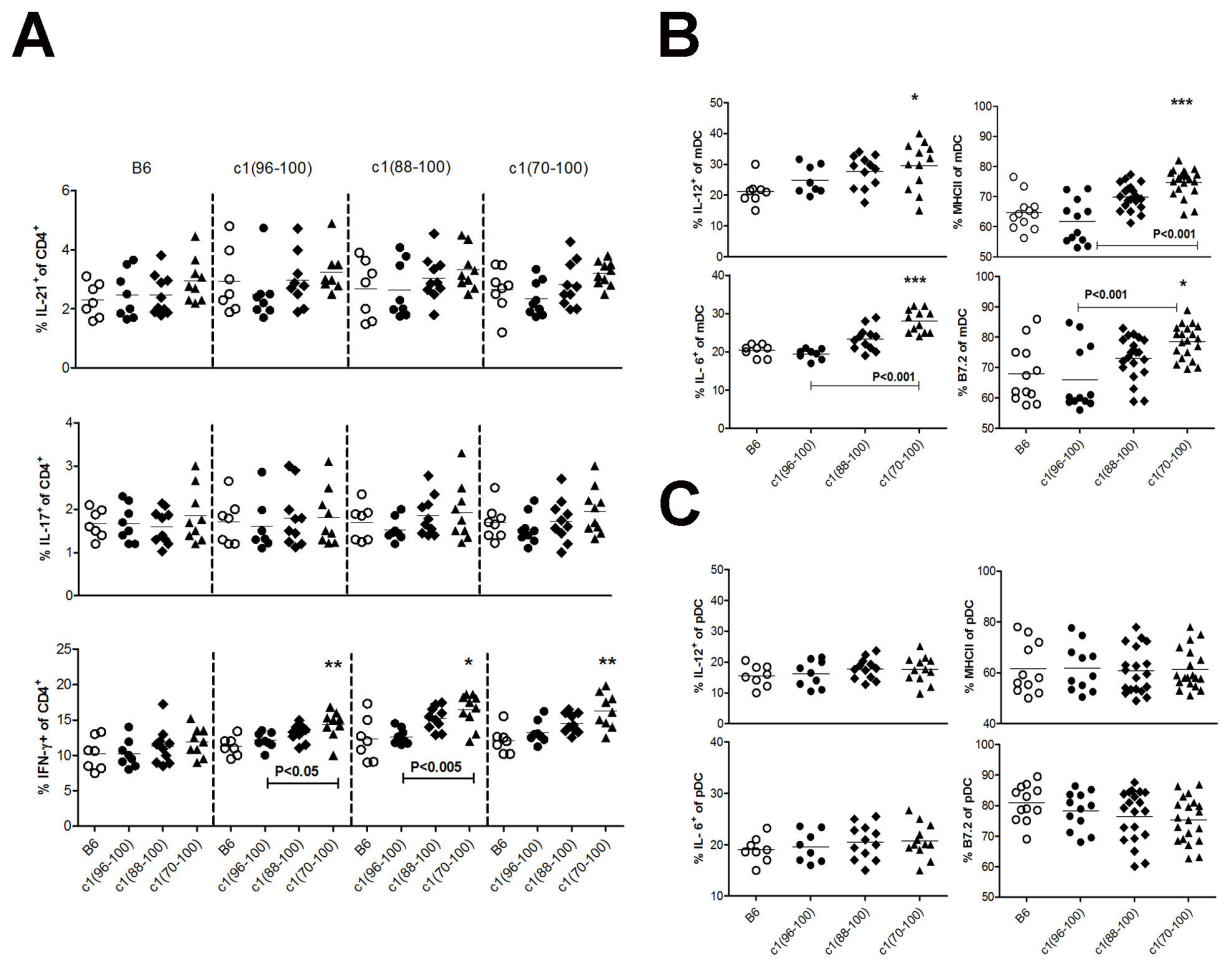
Myeloid DC from c1(88-100) and c1(70-100) mice demonstrate altered function and an enhanced ability to induce differentiation of Th1 cells. BMDC from 8–12 wk-old mice were expanded with FLT3L for 7 days and then co-cultured with OVA peptide and purified naïve CD4^+^ T cells from OT-II TCR Tg mice. On day 4, the cells were re-stimulated with PMA and ionomycin for 4 h in the presence of GolgiStop or GolgiPlug, and analyzed by flow cytometry for cell surface DC (CD11c, CD11b, B220, MHC-II, B7.2) or T cell (CD3, CD4) markers and intracellular cytokine levels. (A) Scatterplots showing the percentage of IL-21-, IL-17- and IFN-γ-producing T cells. Results are clustered in groups based on the strain of T cells (top of the figure) with the DC strain shown at the bottom of the figure. Scatterplots showing the percentage of CD11c^+^CD11b^+^B220^−^ mDC (B) and CD11c^+^CD11b^-^B220^+^ pDC (C) expressing elevated levels of MHCII and B7.2, or IL-6 and IL-12. Results with the different strains of T cells have been pooled as no differences were noted between strains. Horizontal lines indicate the mean. Significance levels were determined by one-way ANOVA with Dunns’ post-test. The *p* values for significant differences between B6 and congenic mouse strains are shown with *p<0.05, **p<0.01, ***p<0.001. Bars with *p* values above denote significant differences between congenic strains.

Cues from DC play an important role in directing the differentiation of T cells following Ag challenge. We therefore contrasted the ability of DC from the various strains of mice to direct the differentiation of OT-II T cells when cultured with low concentrations of OVA *in-vitro*. To this end, bone marrow was isolated from 8-12-wk-old B6 and c1 congenic mice and cultured with FLT3L for 7 days to expand DC. This yielded bone marrow DC (BMDC) that were ~25% plasmacytoid DC (pDC) and ~30% myeloid DC (mDC) with the remaining cells having an indeterminate phenotype. Similar proportions and numbers of DC were seen for all strains (data not shown). The BMDC were then co-cultured with OVA 323-339 peptide and OT-II T cells from B6 or c1 congenic mice for 4 days in the presence of GM-CSF. As shown in Figure 6A, BMDC from c1(70-100) mice demonstrated a significantly enhanced ability to induce differentiation of Th1 cells compared to those from B6 and c1(96-100) mice, and similar non-significant trends were seen for c1(88-100) mice and for Th17 and Tfh cell differentiation. For Th1 cells this increased induction was only seen for OT-II cells from the congenic mouse strains, indicating that T cell and DC defects must interact with each other to induce this phenotype. Similar findings were observed for Th17 cells, where differences between induction of Th17 differentiation by B6 and c1(88-100) or c1(70-100) DC were most pronounced for c1(88-100) and c1(70-100) T cells. Thus, BMDC from congenic mice appear to be able to direct differentiation of T cells in a way that is compatible with the altered differentiation that is observed *in-vivo*. Experiments using BMDC expanded with GM-CSF (data not shown) or whole splenocytes (Figure S4A) as antigen-presenting cells yielded very similar results for comparison of B6 and c1(70-100) cells.

To further explore the mechanisms by which BMDC from c1(88-100) and c1(70-100) mice promote differentiation of pro-inflammatory T cell subsets, mDC activation and cytokine production was examined in the co-culture system. Consistent with their ability to enhance differentiation of Th1 and to a lesser extent Th17 and Tfh cells, BMDC from c1(88-100) and c1(70-100) mice secreted elevated levels of IL-12 and IL-6 which achieved statistical significance for c1(70-100) mice (Figure 6B). Similar findings were seen for c1(70-100) splenic mDC when whole splenoctyes were used as antigen-presenting cells (Figure S4B). A trend to elevated levels of MHC class II and B7.2 were also seen on c1(88-100) mDC, which were further increased on c1(70-100) mDC (Figure 6B). Notably, these changes were independent of the strain of T cells with which the DC were co-cultured (data not shown). In contrast to the data observed for mDC, no differences in cytokine secretion or activation were seen between strains for pDC in the culture (Figure 6C).

**Figure 7:**
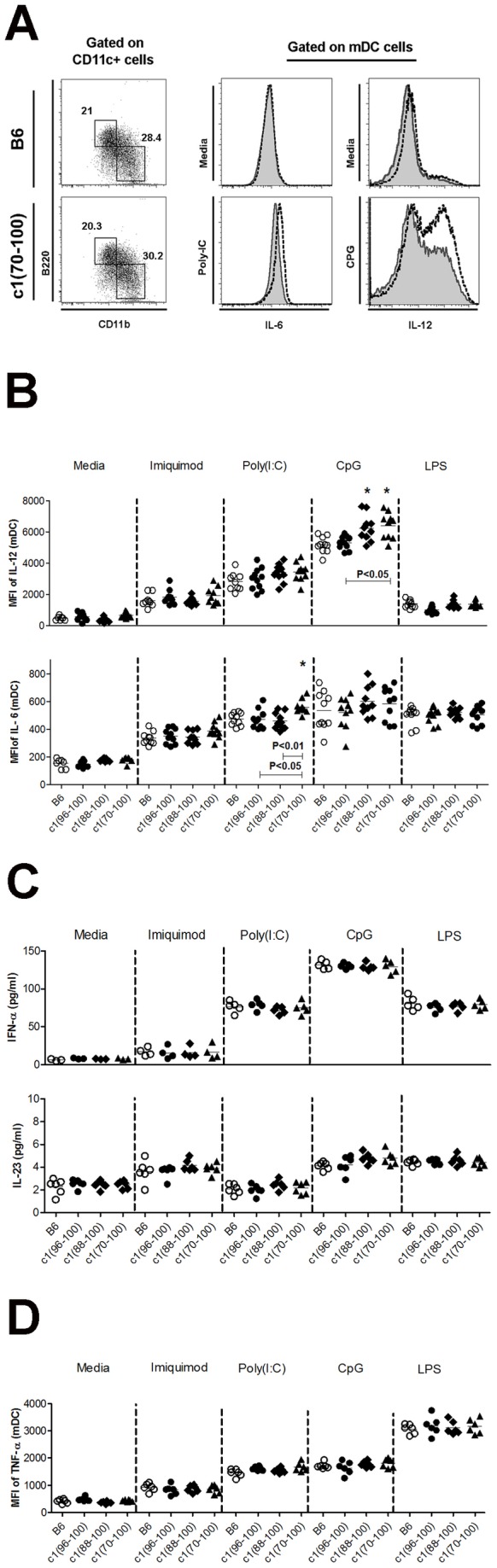
Altered production of IL-6 and/or IL-12 by myeloid DC from c1(88-100) and c1(70-100) mice following stimulation with TLR ligands. BMDC from 8-12 week-old mice were expanded with FLT3L and then cultured in the presence or absence of Imiquimod R837, Poly(I:C), CpG 2216, or LPS for 18h with GolgiStop (for IL-12) or GolgiPlug (for IL-6) being added for the last 6 h. The cells were then stained as outlined in Figure 5 and the Methods. (A) Left panel shows representative dot plots indicating the regions used to gate B220 ^+^ CD11b^-^ pDC (top left box) and B220^-^CD11b^+^ mDC (bottom right box) within the CD11c^+^ population. Shown to the right are representative histogram plots of IL-6 and IL-12 for B6 (solid grey) and c1(70-100) mice (black line) in unstimulated (Media) and stimulated (Poly(I:C) for IL-6 or CpG 2216 for IL-12) conditions. (B) Scatterplots showing the MFI for IL-6 and IL-12 expression on mDC. (C) IFN-α and IL-23 levels in the culture supernatants of BMDC as measured by ELISA. (D) MFI for TNF-α expression in mDC. Horizontal lines indicate the mean. Significance levels were determined by one-way ANOVA with Dunns’ post-test. The *p* values for significant differences between B6 and congenic mouse strains are shown with *p<0.05, **p<0.01. Bars with *p* values above denote significant differences between congenic strains.

In lupus, the immune response is focused on nuclear antigens contained in apoptotic debris. We have previously shown that in NZB c1 congenic mice there is a breach of tolerance to these antigens, resulting in spontaneous priming of histone-reactive T cells [10]. This observation suggests that the DC in these mice may have processed and presented nuclear antigens. Since these nuclear antigens can activate TLRs, enhancing DC activation and presentation, we investigated whether the BMDC abnormality in c1 congenic mice leads to altered TLR responses. Consistent with the results of our co-culture experiments, mDC from c1(88-100) and c1(70-100) mice demonstrated significantly increased intracellular levels of IL-12 and a trend to increased intracellular levels of IL-6 in response to CpG stimulation (Figure 7 A& B). Increased intracellular levels of IL-6 were also observed for c1(70-100) derived mDC following stimulation with Poly(I:C). No differences were seen for the secretion of IFN-α, IL-23 or TNF-α (Figure 7C& D), nor were differences seen for MHC-II or B7.2 expression following TLR stimulation (data not shown). These findings indicate that the altered DC function in c1(88-100) and c1(70-100) mice also affects the response to certain TLR signals.

## Discussion

In this paper, we show that differences in the severity of renal disease that we have previously published for a series of NZB c1 congenic mouse strains correlate with the expansion of pro-inflammatory T cell subsets including, Th1, Th17, and Tfh cells. These findings are compatible with previous work suggesting that these cells populations drive pathogenic autoantibody production and/or inflammatory changes in the kidney [1,2,8,11].

Mice with the shortest NZB c1 interval, c1(96-100), showed no evidence of Tfh cell expansion. Consistent with the lack of Tfh cell expansion, the major increase in cytokine production in these mice appeared to arise from the conventional T cell subset, where slight increases in the number of IFN-γ, IL-17, and IL-21 secreting cells were seen. In contrast, c1(88-100) mice demonstrated significant increases in Tfh and IFN-γ-, IL-17-, and IL-21-producing T cells. While our experiments do not allow us to definitively conclude which expanded cell populations are driving the increased disease severity in these mice, we have previously shown that IgG2a Ab and complement are deposited in their kidneys [9], implicating IFN-γ-producing T cells in this process. However, it is likely that Tfh also play a role, since we have shown that CD40L is necessary for production of GC and nephritis in NZB and c1 mice ( [12] and unpublished observations). Notably, the Tfh cells in c1(88-100) mice do not produce significant amounts of IL-17. This finding contrasts with those observed in BXD2 lupus-prone mice, where substantial numbers of IL-17-producing Tfh cells were seen and introduction of an IL-17R knockout attenuated disease [8].

Although c1(70-100) mice showed trends to increased numbers and/or proportions of IFN-γ-, IL-17-, and IL-21-secreting cells compared to c1(88-100) mice, the most marked differences were for IL-21-secreting cells, particularly those that also secreted IFN-γ (data not shown). Since the severity of renal disease in our mice is closely associated with IgG deposition in the kidney [9], it is likely that these changes augment kidney disease through enhanced selection of pathogenic IgG in the GC. Nevertheless, we cannot exclude a possible role for the IFN-γ or IL-17-producing cells in either providing extra-follicular T cell help or directly impacting inflammation in the kidney in these mice. In keeping with the latter possibility, both IFN-γ- and IL-17-secreting cells have been found infiltrating the kidneys of c1(70-100) mice (unpublished observations).

Experiments in young pre-autoimmune mice immunized with a representative foreign antigen recapitulated the same types of pro-inflammatory cell expansions as seen in older mice. Using various approaches it was demonstrated that alterations in both T cell and DC function contribute to the changes observed. Enhanced differentiation of naïve T cells to IFN-γ-producing cells was localized to the NZB c1 96-100 interval. Although the genetic polymorphism leading to this altered differentiation has not been definitively identified, it is likely that it arises from polymorphisms in the *Slam* family. The NZB c1 96-100 interval overlaps with the region containing the *Sle1b* lupus susceptibility allele identified in NZM2410 mice. NZB, NZM2410, and a variety of other autoimmune mouse strains share the same *Slam* allele which differs from that of B6 mice at genetic loci for multiple *Slam* family members. The top candidate gene in this interval is *Ly108*, which encodes a self-ligating membrane glycoprotein that has at least three alternatively spliced isoforms differing in their cytoplasmic domains [13,14,15]. In autoimmune mouse strains, expression of *Ly108-1* is increased, whereas *Ly108-2* expression is decreased and *Ly108-H1* is absent [14,16]. Since stimulation with anti-Ly108 antibody induces T cell IFN-γ secretion, it is possible that increased expression of the Ly108-1 variant in c1(96-100) T cells leads to enhanced differentiation to IFN-γ-producing cells [17]. Alternatively, the absence of Ly108-H1 expression could produce this phenotype, since introduction of a BAC Ly108-H1 transgene onto mice lacking this isoform was associated with reduced numbers of IFN-γ-producing CD4^+^ T cells *in-vivo* [16]. Recently, B6.*Sle1b* mice were found to have an expansion of Tfh cells that was first detectable at 6-8 months of age (a time point 2 months later than the mice examined in our study) and that could be corrected with the Ly108-H1 transgene [18]. Since c1(96-100) also lack the Ly108-H1 isoform (our own unpublished observations), the findings reported herein suggest that this expansion does not arise from an intrinsic T cell functional defect.

A second T cell functional change, leading to increased generation of IL-17-producing T cells, appeared to require the NZB c1 88-96 interval. It is currently unknown whether this functional change arises solely from genetic polymorphisms located within the c1 88-96 interval or results from interaction between polymorphisms in the c1 96-100 and 88-96 intervals. Candidate genes within the 88-96 interval include: the retinoid X receptor gamma (Rxrg), a member of the RXR family of nuclear receptors which have been shown to modify the balance between Th1 and Th2 cells [19,20,21]; and Pre-B cell leukemia homeobox 1 (*Pbx1*) that has also been shown to influence T cell differentiation. Recently, increased T cell expression of a novel splice isoform of this gene, *Pbx1-d*, was found in B6 congenic mice with the *Sle1a* lupus susceptibility locus [22].

Despite the presence of intrinsic T cell functional abnormalities in c1 congenic mice, this does not appear to be sufficient to induce altered spontaneous T cell differentiation *in vivo*, pointing to a critical role of environmental cues in the induction of the abnormal differentiation of T cells in c1(88-100) and c1(70-100) mice. Nevertheless, T cell abnormalities also appear to be essential, as B6 T cells did not differentiate efficiently to IFN-γ- or IL-17-producing cells following adoptive transfer into c1(70-100) mice *in-vivo*. Our experiments suggest that altered DC function provides one of the environmental cues that enhances pro-inflammatory T cell differentiation in c1(88-100) and c1(70-100) mice. DC from c1(70-100), and to a lesser extent c1(88-100), mice demonstrated enhanced production of IL-12 which has been shown to promote differentiation of naïve T cells to Th1 and Tfh phenotypes [23,24,25] and increased levels of IL-6 which has been shown to promote differentiation of naïve T cells to Th17 and Tfh phenotypes [26,27,28,29]. Following these initial interactions, IL-21 production by activated Th17 and Tfh cells could act in an autocrine manner to further direct DC-primed CD4^+^ T cells to become Th17 and Tfh cells [30,31,32], resulting in a positive feedback loop. It is likely that the enhanced ability of mDC from c1(70-100) and, to a lesser extent, c1(88-100) mice to upregulate MHC class II and B7.2 in response to T-dC interaction further augments the differentiation of pro-inflammatory T cell subsets in these mice.

In summary, we demonstrate that T cell and DC defects, derived from several genetic loci, synergize to convert preclinical disease to fatal GN by leading to expansion of pro-inflammatory T cells. This data joins an increasing body of data from the study of congenic mouse strains demonstrating that impact of individual genetic loci on immune function and autoimmunity is highly dependent upon their genetic/immunologic context [33,34,35,36]. These studies have important implications for the study of human autoimmune disease, in that they provide an explanation for how the presence of a susceptibility locus in the family members of a patient with autoimmune disease can be compatible with relatively normal immune function, whereas the same locus in the patient leads to profoundly altered immune function. Thus, the identification of individuals with an increased likelihood of developing autoimmune disease must necessarily involve characterization of multiple interacting genetic loci.

## Materials and Methods

### Ethics statement

Mice were housed in a Canadian Council on Animal Care approved facility at the Toronto Western Research Institute, part of the University Health Network. All mice used and experiments performed in this study were approved by the Animal Care Committee of the University Health Network (Animal Use Protocol 123).

### Mice

B6 and NZB mice were purchased from Taconic (Germantown, NY) and Harlan Sprague Dawley (Blackthorn, England), respectively. B6.OT-II TCR Tg and B6.Thy1 ^a^IgH^a^ mice were originally obtained from Taconic and The Jackson Laboratory (Bar Harbor, ME), respectively, and bred in our facility. Congenic mice were generated as previously described [9]. OT-II TCR Tg and Thy1 ^a^IgH^a^ (termed Thy1.1 for simplicity) congenic mice were produced by polymorphic marker assisted backcrossing. Only female mice were examined and all mice were specific-pathogen free.

### Flow cytometry

Half a million RBC-depleted splenocytes were incubated with mouse IgG (Sigma-Aldrich) for 15 min prior to staining with various combinations of directly-conjugated mAbs. Allophycocyanin- or PerCP-Cy5.5-conjugated streptavidin (SA) (BD Biosciences) were used to reveal biotin-conjugated Ab staining. Dead cells were excluded by staining with 0.6 µg/ml PI (Sigma-Aldrich). Events were acquired using a LSR II instrument (BD Biosciences) and analyzed using FlowJo software (Tree Star Inc., Ashland, OR). The following directly conjugated mAbs were purchased from BD Biosciences: Biotin-conjugated anti-CXCR5 (2G8), -CD3 (145-2C11), -CD4 (RM4-5), -B220(RA3-6B2), -CD86 (B7.2; GL1), and -CD90.2 (Thy1.2; 30-H12); PE-conjugated anti-CD69 (H1.2F3), -CD44 (IM7), -CD95 (Jo2), -B7.2 (GL1), -IA/IE (M5/114.15.2) and -PD1 (J43); PE-Cy7 conjugated anti-CD44 (IM7) and -CD11c (N418); allophycocyanin-Cy7 conjugated anti-CD44 (IM7) and -CXCR5 (2G8); Pacific Blue-conjugated anti-B220 (RA3-6B2) and -CD4 (RM4-5); PerCP-Cy5.5-conjugated anti-B220 (RA3-6B2); and FITC-conjugated anti-CD90.1 (Thy1.1; OX-7) and -CD11b (M1/70). All isotype controls were obtained from BD Biosciences. Biotin-conjugated peanut agglutinin (PNA) was purchased from Sigma-Aldrich and FITC-conjugated anti-CD62L (MEL-14) mAb was purchased from Cedarlane Laboratories (Burlington, ON, Canada).

### Detection of cytokine-secreting T cells

CD4^+^ T cells were isolated from RBC-depleted splenocytes using the Dynal Mouse CD4 Negative Isolation Kit (114.15D, Invitrogen), re-suspended in complete RPMI medium (10% FBS, non-essential amino acids, L-glutamine, β-mercaptoethanol, penicillin, and streptomycin), and stimulated at 2.5 x 10^5^ cells per well in 96-well plates with plate-bound anti-CD3 Ab (4µg/ml; Cedarlane) and 1 µg/ml soluble anti-CD28 Ab (BD Biosciences). Supernatants were harvested after 72 h and the levels of IL-2, IL-4, IL-17, and IFN-γ measured using a mouse cytometric bead array kit specific for Th1/Th2/Th17 cytokines (BD Biosciences). IL-21 levels were measured using a mouse IL-21 Duo-Set ELISA kit (R&D Systems). Cytokine-secreting CD4^+^ T cells were detected by flow cytometry. RBC-depleted splenocytes were stimulated for 5 h with PMA (50 ng/ml; Sigma-Aldrich) and ionomycin (1 µg/ml; Sigma-Aldrich) in the presence of GolgiStop (BD Biosciences). The cells were stained with Pacific Blue-anti-CD4 and biotinylated-anti-CD3 followed by PerCP-Cy5.5-SA, and then fixed and permeabilized with Cytofix/Cytoperm (BD Biosciences) before intracellular staining with allophycocyanin-anti–IFN-γ (XMG1.2), Alexa Fluor® 488-anti-IL-17A (TC11-18H10), and PE-anti–IL-4 (BVD4-1D11). To quantify IL-21-secreting cells, fixed and permeablized cells were incubated with an IL-21R/Fc chimera (R&D Systems) and then stained with a PE-conjugated affinity-purified F(ab’)_2_ goat anti-human Fc Ab (Jackson ImmunoResearch Laboratories) [37].

### Naïve CD4^+^ T cell isolation and differentiation

Naïve CD4^+^ T cells (CD4^+^CD62L^hi^) were purified using a mouse CD4^+^CD62L^+^ T Cell Isolation Kit (Miltenyi Biotec), re-suspended in complete RPMI, and stimulated with plate-bound anti-CD3 (4 µg/ml) and soluble anti-CD28 (1 µg/ml) in 96-well plates under the following conditions (all cytokines and mAb purchased from R&D Systems): Th0 cell: anti-IFN-γ (10 µg/ml) and anti-IL-4 (10 µg/ml); Th1 cell: IL-12 (10 ng/ml), and anti-IL-4 (10 µg/ml); Th2 cell: IL-4 (10 ng/ml), and anti-IFN-γ (10 µg/ml); Th17 cell: IL-6 (10 ng/ml), IL-23 (10 ng/ml), TGF-β1 (2.5 ng/ml), anti-IFN-γ (10 µg/ml) and anti-IL-4 (10 µg/ml); and IL-21-producing T cell: IL-6 (30 ng/ml), anti-IFN-γ (10 µg/ml) and anti-IL-4 (10 µg/ml). After 4 days, the cells were washed and re-stimulated for 4 h with 50 ng/ml PMA and 1 µg/ml ionomycin in the presence of GolgiStop. Cytokine-secreting T cells were quantified as outlined previously.

### In-vivo differentiation of OVA-specific T cells

Mice (8-12-wk old) were immunized i.p. with 100 µg OVA (Grade II, Sigma) or PBS emulsified in CFA (Sigma) and sacrificed 2 wks later. For measurement of OVA-specific cytokine production, 1 x 10^6^ RBC-depleted splenocytes were cultured in complete RPMI (5% FBS) alone, or containing 100 µg/ml OVA, per well in 96-well plates. Supernatants were harvested at 72 h and assayed for IL-4, IFN-γ, IL-17, and IL-21, as outlined previously. For adoptive transfers, 3 x 10^6^ naive splenic CD4^+^ T cells from 8-10-wk-old B6 or congenic OT-II mice were injected into the tail vein of 8-10-wk-old B6.Thy1.1 or c1(70-100). Thy1.1 recipients. The following day mice were immunized with OVA emulsified in CFA. The proportion of various T cell subsets within the spleen was determined 2 wks later by flow cytometry after gating Thy1.2^+^ (transferred) T cells.

### Immunofluorescence staining of tissue sections

Spleens were snap-frozen in OCT compound (Sakura Finetek, Torrance, CA) at the time of sacrifice. Cryostat sections (5 µm) were fixed in acetone, washed with PBS, and blocked with 5% FBS/PBS. Sections were stained with biotinylated-PNA, allophycocyanin-conjugated anti-CD4, PE-conjugated anti-PD1 and FITC-conjugated anti-IgM F(ab’)_2_ (Jackson Immunoresearch), to detect Tfh cells within GC. Biotin staining was revealed using 7-amino-4-methylcoumarin-3-acetic acid-conjugated streptavidin as a secondary reagent (Jackson Immunoresearch). Stained sections were mounted with Fluoro-Gel (Electron Microscopy Sciences) and tissue fluorescence was visualized using a Zeiss Axioplan 2 imaging microscope (Oberkochen, Germany). Digital images were obtained using the manufacturer’s imaging system.

### BMDC isolation and stimulation

Bone marrow cells were isolated by flushing femurs of 8–12 wk-old mice. After RBC lysis, the cells were re-suspended at 10^6^ cells/mL and cultured for 7 days with recombinant human FLT3L (20 ng/mL; R&D Systems) in complete RPMI. For TLR stimulation, 4 x 10^5^ cells were cultured in 96-well flat-bottom plates for 24 h with media alone or containing various TLR ligands including: Imiquimod (2 µM), Poly(I:C) (50 µg/mL), CpG ODN 2216 or control (250 nM), CpG ODN 1826 or control (250 nM) (all from InvivoGen, San Diego, CA), or LPS (25 µg/mL; Sigma-Aldrich), as a positive control. The cells were then harvested and stained with anti-CD11c, -CD11b, and -B220. Staining with anti-CD86 (B7.2) and -MHC-II was used to assess cellular activation and intra-cellular levels of IL-12, IL-6 and TNF-α were used to assess cytokine production. GolgiStop or GolgiPlug (BD Biosciences) were added to cell cultures for the last 4 h of the incubation, prior to measurement of intracellular cytokines, which were detected using allophycocyanin-conjugated anti-IL-12 (C15.6), Alexa Fluor® 488-conjugated anti-IL-6 (MP5-20F3) and PE-conjugated anti–TNF-α (TN3-19.12). BD Horizon fixable viability station 450 (FVS450) was used to exclude dead cells. To assess IFN-α and IL-23 production, cytokine levels in tissue culture supernatants were measured by ELISA kits as follows: IFN-α (PBL Biomedical Laboratories, Piscataway, NJ); and IL-23 (IL-23 Duo-Set, R&D Systems).

### In-vitro culture of BMDCs and OVA-specific T cells

2x10^4^ BMDC were co-cultured with OVA 323-339 peptide (GenScript, Piscataway, NJ) and 2x10^5^ naïve CD4^+^ T cells, isolated from the spleens of 8-10-wk-old B6.OT-II or c1 congenic OT.II mice, in the presence of 5 ng/ml recombinant mouse GM-CSF (R&D Systems) for 4 days. Cells were stimulated with PMA (50 ng/ml) and ionomycin (1 µg/ml) in the presence of GolgiPlug or GolgiStop (BD Biosciences) for 4 h before harvesting. The cells were then stained for cell surface DC (CD11c, CD11b, B220) or T cell (CD3, CD4) markers, fixed, permeabilized, and stained for detection of intracellular cytokines, including IL-6, IL-12, IL-21, IL-17 and IFN-γ, as outlined previously.

### In-vitro culture of splenocytes and OVA-specific T cells

Splenocytes were isolated from 5-6-wk-old B6.Thy1.1 or c1(70-100). Thy1.1 mice. Total splenocytes were seeded in 96-well U-bottom plates at 2x10^5^ cells per well, then co-cultured for 72 hr with 1 µg/ml OVA 323-339 peptide (GenScript, Piscataway, NJ) and 2x10^5^ purified naïve CD4^+^ T cells isolated from the spleens of 8-10-wk-old B6.OT-II or c1(70-100) congenic OT.II mice. PMA (50 ng/ml) and ionomycin (1 µg/ml) together with GolgiPlug or GolgiStop (BD Biosciences) were added for the last 4 h before harvesting. The cells were then stained for cell surface DC (CD11c, CD11b, B220), B cells (CD19 and B220) or T cell (CD3, CD4) markers, fixed, permeabilized, and stained for detection of intracellular cytokines, as outlined previously.

### Statistical analysis

Comparisons of differences between groups of mice for continuous data were performed using one-way ANOVA followed by Dunns’ post-test for multiple comparisons. All statistical analyses were performed using GraphPad software (La Jolla, CA, USA).

## Supporting Information

Figure S1 **c1 congenic mice have an increased proportion of GC B and Tfh cells.** Freshly isolated splenocytes from 4-mo-old B6, c1(96-100), c1(88-100), and c1(70-100) mice were stained with anti-B220 in combination with anti-Fas and PNA to assess the proportion of splenic GC B cells (B220 ^+^ Fas ^+^ PNA^hi^). (A) Shown are contour plots gated on PI-excluding splenocytes from B6 and c1(70-100) mice. Boxes indicate the regions that were used to define GC B cells, with the numbers above them indicating the proportion of cells in the gated population. (B) Scatterplot showing the proportions of GC B cells in the various mouse strains. Each point represents the determination from an individual mouse. Horizontal lines indicate the mean of each group examined. (C) Splenic sections from 4 month old B6, c1(96-100), c1(88-100) and c1(70-100) mice were stained with FITC anti-IgM (Green), biotinylated PNA followed by 7-amino-4-methylcoumarin-3-acetic acid-conjugated streptavidin (Blue), PE anti-PD1 (Yellow) and allophycocyanin anti-CD4 (Purple). Arrows indicate the location of Tfh cells within the germinal center for each mouse strain. Note the increased numbers of Tfh cells (white dots) distributed throughout the large germinal center in c1(70-100) and to a lesser extent c1(88-100) mice. Magnification= ✕ 10. The scale bar indicates 100 µm. (D) Scatter plot showing the number of Tfh cells within GC. Each point represents the average number of Tfh cells per GC for an individual mouse, with 5-7 GC being counted per mouse. Horizontal lines indicate the mean of each group examined. Significance levels were determined by one-way ANOVA with Dunns’ post-test. The *p* values for significant differences between B6 and congenic mouse strains are shown with *p<0.05, **p<0.01, ***p<0.001. Bars with *p* values above denote significant differences between congenic strains.
(TIF)

Figure S2 **c1 congenic mice exhibit increased production of cytokines secreted by Tfh, Th1 and Th17 populations.** Splenic CD4^+^ T cells were purified from 4-mo-old B6, c1(96-100), c1(88-100), and c1(70-100) mice using negative selection and were cultured with plate-bound anti-CD3 antibody in the presence of anti-CD28 for 48 h. Culture supernatants were assayed for cytokine production in triplicate with the levels of IL-2, IL-4, IL-17, and IFN-γ being determined using a cytokine bead array, and for IL-21 by ELISA. Each point represents the determination from an individual mouse. Horizontal lines indicate the mean for each population examined. Significance levels were determined by one-way ANOVA with Dunns’ post-test. The *p* values for significant differences between B6 and congenic mouse strains are shown with *p<0.05, **p<0.01, ***p<0.001. Bars with *p* values above denote significant differences between congenic strains.
(TIF)

Figure S3 **Identification of cytokine-producing T cell subsets in c1 congenic mice.** Freshly isolated splenocytes from 4-mo-old mice were stained with anti-CD3, -CD4, -CXCR5, and -PD1, permeabilized and then stained for intracellular IL-17 and IFN-γ production (as described in Figure 2) with the addition of IL-21R/Fc chimera to detect IL-21 production. (A) Representative contour plots gated on CD3^+^CD4^+^ T cells from B6 and c1(70-100) mice are shown on the left for each strain. The regions used to define the Tfh and conventional (non-Tfh) cells are shown. Numbers indicate the proportion of each cell subset in the gated population. To the right are contour plots showing representative results for cytokine staining. The quadrants used to identify positively staining cells are shown. (B) Scatterplots showing the absolute number of Tfh, and non-Tfh cells producing IL-21 (top), IL-17 (middle), and IFN-γ (bottom). Each point represents the determination from an individual mouse. Horizontal lines indicate the mean for each population examined. (C) Splenic sections from 4-mo-old B6, c1(96-100), c1(88-00), and c1(70-100) mice were stained with FITC anti-IgM (Green), biotinylated-PNA followed by 7-amino-4-methylcoumarin-3-acetic acid-conjugated streptavidin (Blue), PE anti-IL-17 (Yellow) and allophycocyanin anti-CD4 (Purple). Arrows indicate the location of IL-17 producing CD4^+^ T cells within T cell areas for each mouse strain. Note that the increased numbers of IL-17-producing CD4^+^ T cells (white dots) in c1(70-100) mice are located predominantly in the T cell zone and not the GC. Magnification = ∉ 10. The scale bar indicates 100 µm. (D) Scatter plot showing the number of IL-17-producing CD4^+^ T cells within the T cell zone. Each point represents the average number of IL-17-producing cells per T cell zone for an individual mouse, with 5-7 T cell zones being counted per mouse. Significance levels were determined by one-way ANOVA with Dunns’ post-test. The *p* values for significant differences between B6 and congenic mouse strains are shown with *p<0.05, **p<0.01, ***p<0.001. Bars with *p* values above denote significant differences between congenic strains.
(TIF)

Figure S4 **Splenic mDC from c1(70-100) congenic showed increased production of IL-6 and IL-12, and induce enhanced T cell differentiation *in-vitro*.** Freshly isolated splenocytes from 5-6-wk old B6.Thy1.1 or c1(70-100). Thy1.1 were co-cultured with OVA peptide and purified naïve CD4^+^ T cells from OT-II TCR Tg B6 and c1(70-100) mice. On day 3, the cells were re-stimulated with PMA and ionomycin for 4 h in the presence of GolgiStop or GolgiPlug, and analyzed by flow cytometry for cell surface DC (CD11c, CD11b, B220), B cell (CD19, B220) or T cell (CD3, CD4) markers and intracellular cytokine levels. (A) Scatterplots showing the percentage of IL-21-, IL-17- and IFN-γ-producing T cells. Results are clustered in groups based on the strain of the T cells (top of the figure) with the strain of origin of the splenocytes shown at the bottom of the figure. (B) Scatterplots showing the percentage of B cells and mDC producing IL-12 and IL-6. (C) Scatterplot showing the proportion of B cells and mDC within the splenic population. Horizontal lines indicate the mean.
(TIF)
