# Supplementary figures and images for: T Cell and Dendritic Cell Abnormalities Synergize to Expand Pro-Inflammatory T Cell Subsets Leading to Fatal Autoimmunity in B6.NZBc1 Lupus-Prone Mice
Source: PLoS One. 2013 Sep 20;8(9):e75166. doi: 10.1371/journal.pone.0075166 (PMC3779178; doi:10.1371/journal.pone.0075166)

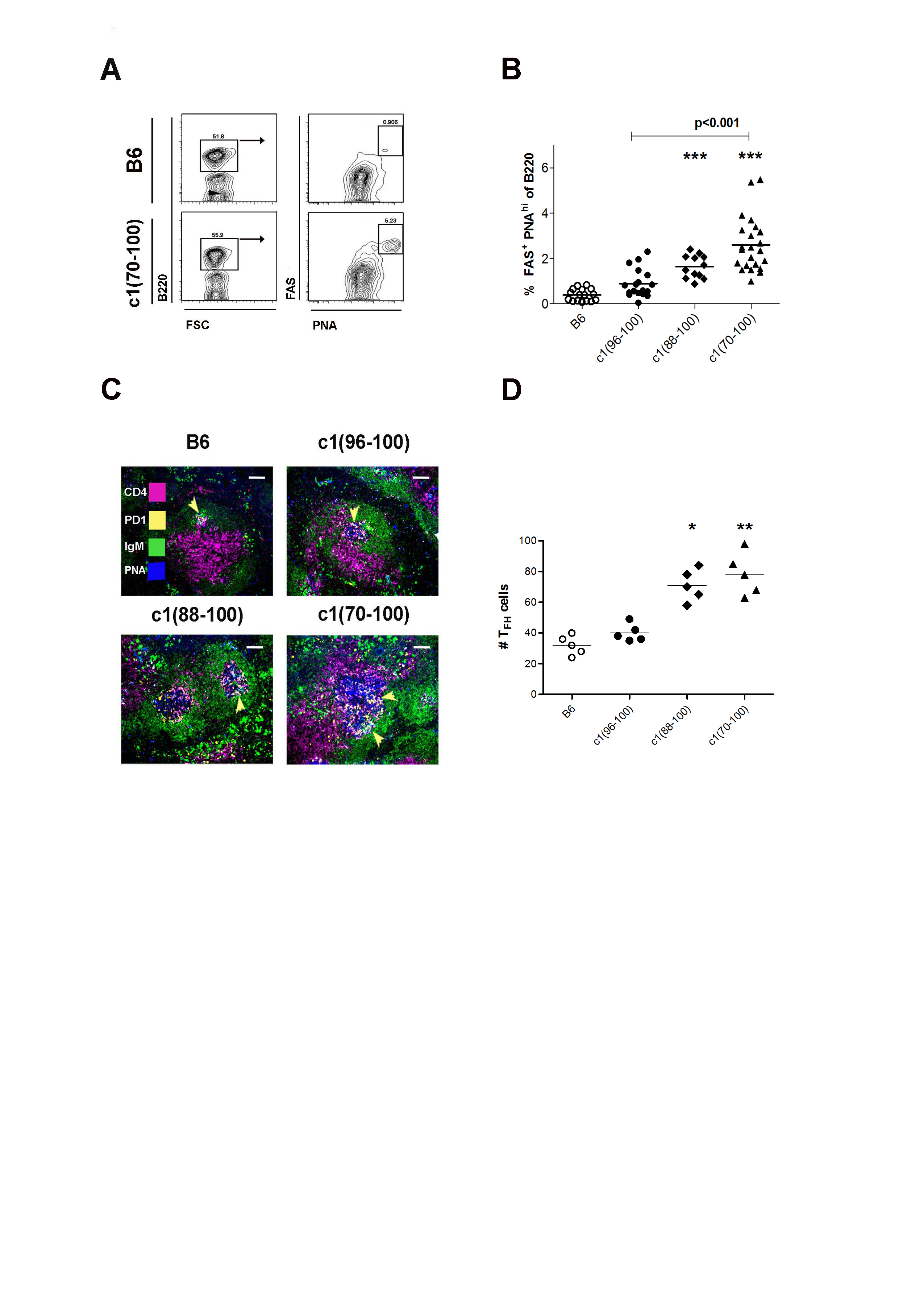

Supplement: Figure S1 — c1 congenic mice have an increased proportion of GC B and Tfh cells. Freshly isolated splenocytes from 4-mo-old B6, c1(96-100), c1(88-100), and c1(70-100) mice were stained with anti-B220 in combination with anti-Fas and PNA to assess the proportion of splenic GC B cells (B220 + Fas + PNAhi). (A) Shown are contour plots gated on PI-excluding splenocytes from B6 and c1(70-100) mice. Boxes indicate the regions that were used to define GC B cells, with the numbers above them indicating the proportion of cells in the gated population. (B) Scatterplot showing the proportions of GC B cells in the various mouse strains. Each point represents the determination from an individual mouse. Horizontal lines indicate the mean of each group examined. (C) Splenic sections from 4 month old B6, c1(96-100), c1(88-100) and c1(70-100) mice were stained with FITC anti-IgM (Green), biotinylated PNA followed by 7-amino-4-methylcoumarin-3-acetic acid-conjugated streptavidin (Blue), PE anti-PD1 (Yellow) and allophycocyanin anti-CD4 (Purple). Arrows indicate the location of Tfh cells within the germinal center for each mouse strain. Note the increased numbers of Tfh cells (white dots) distributed throughout the large germinal center in c1(70-100) and to a lesser extent c1(88-100) mice. Magnification= ✕ 10. The scale bar indicates 100 µm. (D) Scatter plot showing the number of Tfh cells within GC. Each point represents the average number of Tfh cells per GC for an individual mouse, with 5-7 GC being counted per mouse. Horizontal lines indicate the mean of each group examined. Significance levels were determined by one-way ANOVA with Dunns’ post-test. The p values for significant differences between B6 and congenic mouse strains are shown with *p<0.05, **p<0.01, ***p<0.001. Bars with p values above denote significant differences between congenic strains. (TIF) [file pone.0075166.s001.tif]

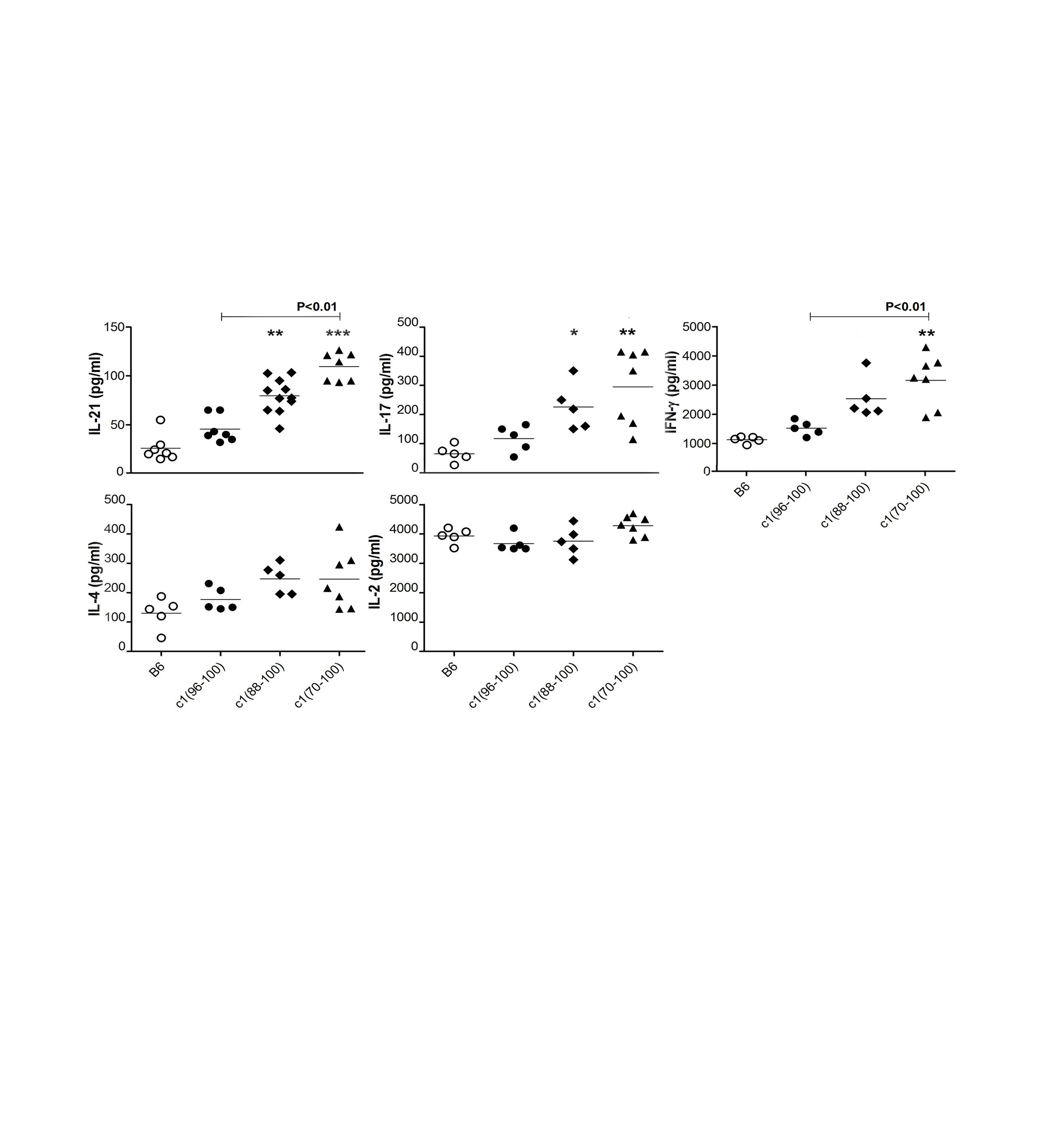

Supplement: Figure S2 — c1 congenic mice exhibit increased production of cytokines secreted by Tfh, Th1 and Th17 populations. Splenic CD4+ T cells were purified from 4-mo-old B6, c1(96-100), c1(88-100), and c1(70-100) mice using negative selection and were cultured with plate-bound anti-CD3 antibody in the presence of anti-CD28 for 48 h. Culture supernatants were assayed for cytokine production in triplicate with the levels of IL-2, IL-4, IL-17, and IFN-γ being determined using a cytokine bead array, and for IL-21 by ELISA. Each point represents the determination from an individual mouse. Horizontal lines indicate the mean for each population examined. Significance levels were determined by one-way ANOVA with Dunns’ post-test. The p values for significant differences between B6 and congenic mouse strains are shown with *p<0.05, **p<0.01, ***p<0.001. Bars with p values above denote significant differences between congenic strains. (TIF) [file pone.0075166.s002.tif]

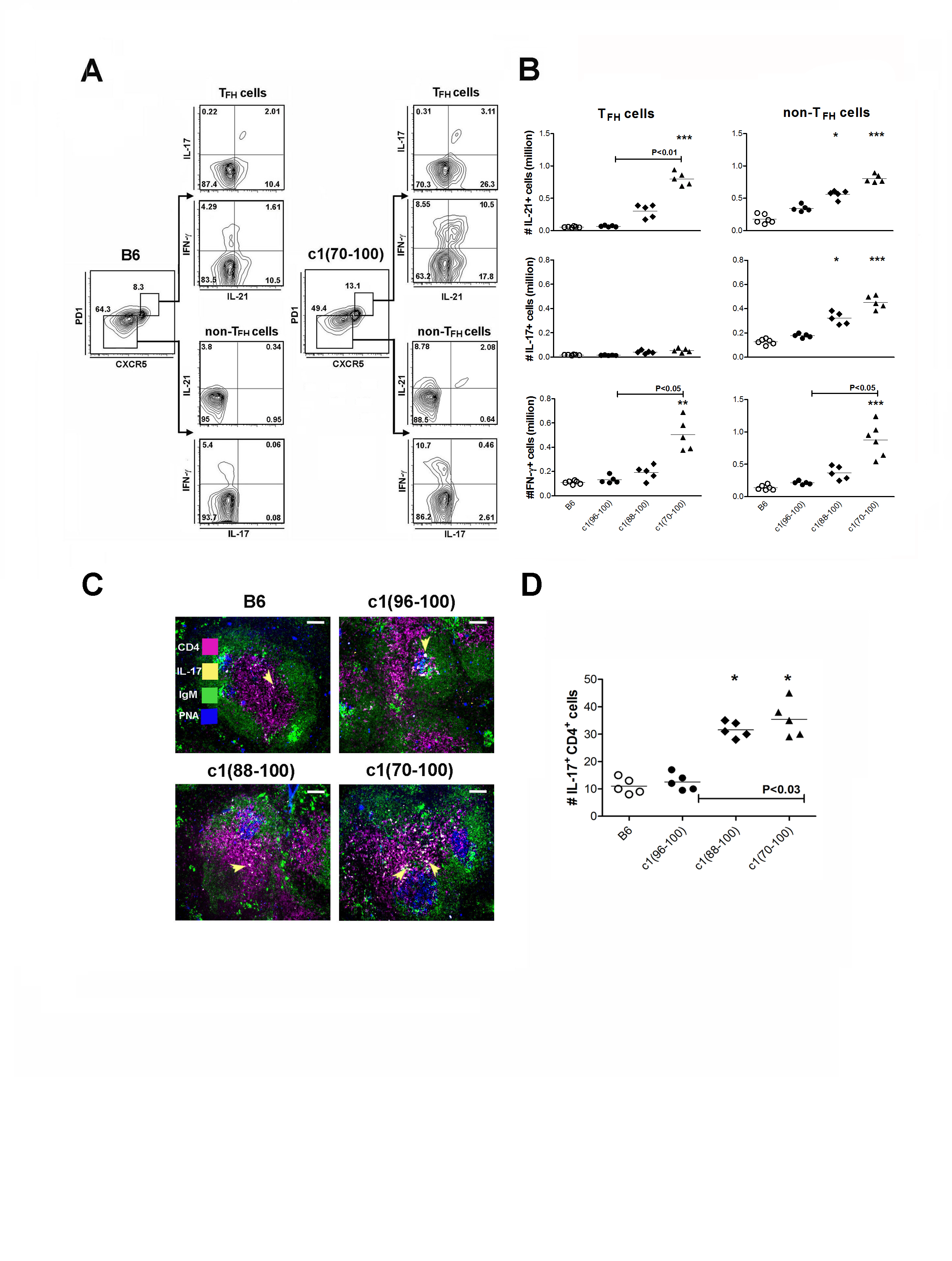

Supplement: Figure S3 — Identification of cytokine-producing T cell subsets in c1 congenic mice. Freshly isolated splenocytes from 4-mo-old mice were stained with anti-CD3, -CD4, -CXCR5, and -PD1, permeabilized and then stained for intracellular IL-17 and IFN-γ production (as described in Figure 2) with the addition of IL-21R/Fc chimera to detect IL-21 production. (A) Representative contour plots gated on CD3+CD4+ T cells from B6 and c1(70-100) mice are shown on the left for each strain. The regions used to define the Tfh and conventional (non-Tfh) cells are shown. Numbers indicate the proportion of each cell subset in the gated population. To the right are contour plots showing representative results for cytokine staining. The quadrants used to identify positively staining cells are shown. (B) Scatterplots showing the absolute number of Tfh, and non-Tfh cells producing IL-21 (top), IL-17 (middle), and IFN-γ (bottom). Each point represents the determination from an individual mouse. Horizontal lines indicate the mean for each population examined. (C) Splenic sections from 4-mo-old B6, c1(96-100), c1(88-00), and c1(70-100) mice were stained with FITC anti-IgM (Green), biotinylated-PNA followed by 7-amino-4-methylcoumarin-3-acetic acid-conjugated streptavidin (Blue), PE anti-IL-17 (Yellow) and allophycocyanin anti-CD4 (Purple). Arrows indicate the location of IL-17 producing CD4+ T cells within T cell areas for each mouse strain. Note that the increased numbers of IL-17-producing CD4+ T cells (white dots) in c1(70-100) mice are located predominantly in the T cell zone and not the GC. Magnification = ∉ 10. The scale bar indicates 100 µm. (D) Scatter plot showing the number of IL-17-producing CD4+ T cells within the T cell zone. Each point represents the average number of IL-17-producing cells per T cell zone for an individual mouse, with 5-7 T cell zones being counted per mouse. Significance levels were determined by one-way ANOVA with Dunns’ post-test. The p values for significant difference [file pone.0075166.s003.tif]

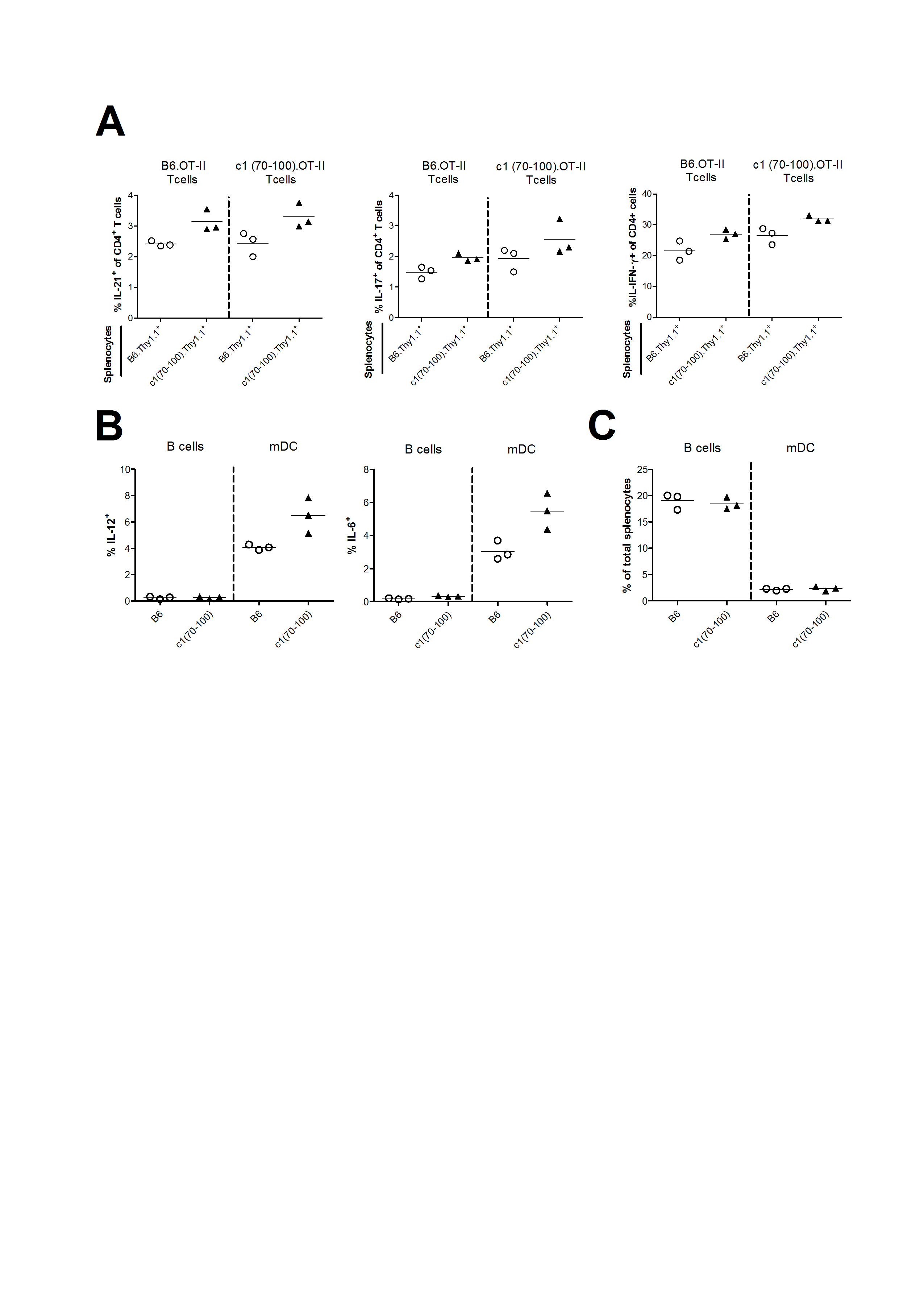

Supplement: Figure S4 — Splenic mDC from c1(70-100) congenic showed increased production of IL-6 and IL-12, and induce enhanced T cell differentiation in-vitro. Freshly isolated splenocytes from 5-6-wk old B6.Thy1.1 or c1(70-100). Thy1.1 were co-cultured with OVA peptide and purified naïve CD4+ T cells from OT-II TCR Tg B6 and c1(70-100) mice. On day 3, the cells were re-stimulated with PMA and ionomycin for 4 h in the presence of GolgiStop or GolgiPlug, and analyzed by flow cytometry for cell surface DC (CD11c, CD11b, B220), B cell (CD19, B220) or T cell (CD3, CD4) markers and intracellular cytokine levels. (A) Scatterplots showing the percentage of IL-21-, IL-17- and IFN-γ-producing T cells. Results are clustered in groups based on the strain of the T cells (top of the figure) with the strain of origin of the splenocytes shown at the bottom of the figure. (B) Scatterplots showing the percentage of B cells and mDC producing IL-12 and IL-6. (C) Scatterplot showing the proportion of B cells and mDC within the splenic population. Horizontal lines indicate the mean. (TIF) [file pone.0075166.s004.tif]
